# The whole mitochondrial genome and phylogenetic analysis of *Lupocycloporus gracilimanus* (Stimpson, 1858) (Decapoda, portunidae)

**DOI:** 10.1080/23802359.2018.1532345

**Published:** 2018-10-26

**Authors:** Mengyun Guan, Huaqiang Tan, Hanafiah Fazhan, Zhuofang Xie, Xi Shi, Yin Zhang, Fan Lin, Mhd Ikhwanuddin, Hongyu Ma

**Affiliations:** a Guangdong Provincial Key Laboratory of Marine Biotechnology, Shantou University, Shantou, China;; b STU-UMT Joint Shellfish Research Laboratory, Shantou University, Shantou, China;; c Institute of Tropical Aquaculture, Universiti Malaysia Terengganu, Kuala Terengganu, Malaysia

**Keywords:** *Lupocycloporus gracilimanus*, mitochondrialgenome, phylogeny

## Abstract

The mitochondrial genome plays an important role in studies on phylogeography and population genetic diversity. Here we report the complete mitochondrial genome of *Lupocycloporus gracilimanus* (Stimpson, 1858) which is the first mitochondrial genome reported in genus *Lupocycloporus* by now. The mitogenome is 15,990 bp in length, consisting of 13 protein-coding genes, 22 transfer RNA genes, two ribosomal RNA genes and a putative control region. The phylogenetic analysis showed that *L. gracilimanus* was closest to genus *Scylla*. The present research should provide valuable information for phylogenetic analysis and classification of Portunidae.

The small hand swimming crab, *Lupocycloporus gracilimanus* (Stimpson, 1858), is under Portunidae. It is mainly distributed in coasts of China, Australia, New Zealand, Philippines, Malaysia, Andaman (Dai et al. 1986), and also recorded along the west coast of the Indian Ocean in 2005 (Dineshbabu 2005). Additionally, *L. gracilimanus* is one of the dominant crab species in autumn in East China Sea (Yu et al. 2005). So far, limited information about the phylogeny and genetic diversity are available. To well-understand the evolutionary status of *L. gracilimanus* and their position in family Portunidae, we amplified and sequenced the complete mitochondrial genome of *L. gracilimanus*.

Specimens of *L. gracilimanus* were collected from Weizhou Island (21.0234N, 109.0940E), Guangxi province of China, and deposited at the Marine Biology Institute of Shantou University, Shantou of China. Genomic DNA was extracted from muscle tissue using traditional phenol–chloroform method. Long and conventional PCR methods were applied to obtain the whole mitogenome sequence. The mitochondrial genome size of *L. gracilimanus* is 15,990 bp (GenBank accession number: MH729187), including 13 protein-coding genes, 22 transfer RNA genes, two ribosomal RNA genes and a putative control region. The composition and order of genes were consistent with other species such as *Portunus* (Yamauchi et al. 2003; Ma et al. 2013) and *Charybdis* (Ma et al. 2015; Yang et al. 2017). The overall mitogenome composition is 34.05% for A, 11.23% for G, 19.02% for C and 35.70% for T, respectively. Among all the 37 genes, 23 were encoded by heavy strand and 14 were encoded by light strand. Three types of initiation codons were found in the 13 protein-coding genes, including ATG (COX1, COX2, ATP8, COX3, ND5, ND4, ND4L, ND6, Cytb and ND2 gene), ATT (ATP6 and ND3 gene) and ATA (ND1 gene). Four kinds of termination codons including two incomplete termination codons (T-, TAG, TA- and TAA) were also found.

In order to analyze the phylogenetic position of *L. gracilimanus*, 12 protein-coding genes (except ND6) from 16 crab species in GenBank database were used to construct the phylogenetic tree by maximum-likelihood (ML) method (Figure 1). *Panulirus japonicus* was served as an outgroup for tree rooting. The result showed that *L. gracilimanus* was genetically closer to genus *Scylla* (*S. paramamosain*, *S. tranquebarica*, *S. serrata* and *S. olivacea*) than to genus *Portunus* (*P. sanguinolentus*, *P. trituberculatus* and *P. pelagicus*). This is consistent with the previous research by Evans (2018). Moreover, *Lupocycloporus* was recommended as a genus in subfamily Lupocyclinae according to their morphological similarity (Spiridonov et al. 2014). This study should provide valuable data for phylogenetic analysis and classification for Portunidae.

**Figure 1.**
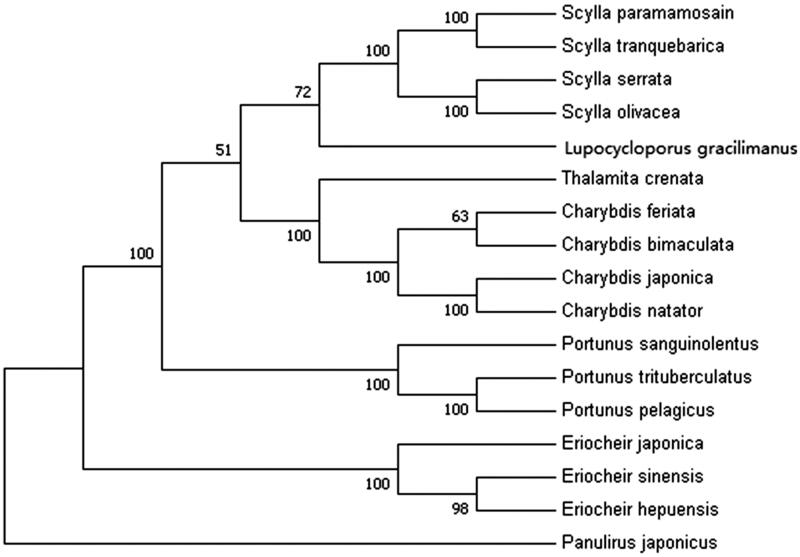
Phylogenetic tree of *Lupocycloporus gracilimanus* and related species based on maximum-likelihood (ML) method. *Panulirus japonicus* was served as an outgroup.
